# Surgical Treatment on Subungual Osteochondromas in Paediatric Feet: A Case Series Study

**DOI:** 10.3390/jcm9041122

**Published:** 2020-04-14

**Authors:** Emmanuel Navarro-Flores, Daniel López-López, Ricardo Becerro-de-Bengoa-Vallejo, Marta Elena Losa-Iglesias, Carlos Romero-Morales, Marta San Antolín-Gil, César Calvo-Lobo, Adolfo Laureano Bautista-Casasnovas

**Affiliations:** 1 Frailty Research Organizaded Group (FROG), Faculty of Nursing and Podiatry, Department of Nursing, University of Valencia, 46010 Valencia, Spain; emmanuel.navarro@uv.es; 2 Research, Health and Podiatry Group, Department of Health Sciences, Faculty of Nursing and Podiatry, Universidade da Coruña, 15403 Ferrol, Spain; 3 School of Nursing, Physiotherapy and Podiatry, Universidad Complutense de Madrid, 28040 Madrid, Spain; ribebeva@ucm.es (R.B.-d.-B.-V.); cescalvo@ucm.es (C.C.-L.); 4 Faculty of Health Sciences, Universidad Rey Juan Carlos, 28922 Alcorcón, Spain; marta.losa@urjc.es; 5 Faculty of Sport Sciences, Universidad Europea de Madrid, Villaviciosa de Odón, 28670 Madrid, Spain; carlos.romero@universidadeuropea.es; 6 Department of Psychology, Universidad Europea de Madrid, Villaviciosa de Odón, 28670 Madrid, Spain; marta.sanantolin@universidadeuropea.es; 7 Department of Pediatric Surgery, Hospital Clínico Universitario de Santiago, 15706 Santiago de Compostela, Spain; adolfo.bautista@usc.es

**Keywords:** paediatric foot, subungual osteochondroma, children, bone tumour

## Abstract

Subungual osteochondroma (SO) is an infrequent and non-malignant bone tumour of the distal phalanx, especially prominent in paediatric populations. The aim of this research was to describe a case series of paediatric feet with SO which received surgical treatments. The secondary purpose was to compare these descriptive data by sex distribution. Methods: Twenty-three paediatric feet with SO confirmed by clinical or radiological features received surgical treatment. Socio-demographic (age, sex, height, weight and BMI) and clinical features (side, location, tumour or pain presence, and nail lift before surgery, as well as recurrence and adverse effects at one month after intervention) were reported. Results: Regarding clinical features before intervention, the most frequent locations of SO were the first toe (86.8%) and the right lower limb (56.5%). In addition, the presence of the tumour, pain and nail lift showed a prevalence of 91.3%, 69.5% and 47% of the study sample, respectively. Considering clinical features at one month after intervention, the most frequent adverse effect was the pain presence (69.5%). In addition, one case (4.4%) presented ulceration. Only one patient (4.4%) suffered from recurrence with a new tumour. There were not statistically significant differences by sex distribution (*p* > 0.05). Conclusions: This novel study showed that surgery treatment for SO in paediatric populations presented a very low recurrence degree with minor adverse effects and without differences by sex distribution. Thus, further randomized clinical trials should be carried out in order to determine the effectiveness of this intervention in this special population.

## 1. Introduction

Subungual osteochondroma (SO), also named exostosis, is a projection bone within cartilage covering. Studies have shown that an isolated osteochondroma may be the most common presentation (90%), although it may also be presented as genetic multi exostoses [1,2]. Frequently, symptoms appear after several months of this presentation, including pain, erythema, and deformity of the nail bed [3,4]. 

The main clinical sign is a firm and fixed nodule with a hyperkeratotic surface at the distal extreme of the nail plate. A delayed diagnosis of the lesion seems to be common, therefore appropriate treatment is not often rendered [5]. 

This condition is the most common non-malignant bone tumour and mainly affects pubescent and young adults, with a predilection in males [3,6,7,8,9,10,11]. Solitary exostosis represents 50% of initial bone neoplasms and 15% of all bone neoplasm [12]. In osteochondromas, bone appears from endochondral ossification, where bone develops from fibrous tissue in subungual exostosis [1,2].

This condition may have a negative impact on health-related quality of life [13]. In addition, SO may be considered as a significant problem for foot health, given its high incidence, which is related to foot surgery, and its association with dermatological symptoms.

Osteochondroma usually appears in lower limbs placed in distal areas, it represents a rare diagnosis and needs a differential diagnosis of subungual exostosis. This diagnosis may be carried out using clinical, radiological, and histological criteria, considering excisional surgery as a first line intervention [6,7,14]. Osteochondromas are often asymptomatic and may frequently escape detection procedures, being mainly located in the metaphysis, and most osteochondromas are localized in the distal phalanx [1,3,15,16]. 

In children, this condition represents a low percentage as SO is not often manifested clinically, and, consequently, case series reporting this disorder and procedure need to be detailed [1,6]. Thus, the aim of this research was to describe a case series of paediatric feet with SO which received surgical treatments. The secondary purpose was to compare these descriptive data by sex distribution.

## 2. Materials and Methods

### 2.1. Design and Sample

The research was a case series study developed in Complexo Hospitalario de Santiago de Compostela (CHUS), Spain and Policlínico Vigo, Spain, between May 2014 and December 2019, according to the Consensus-Based Clinical Case Reporting Guideline Development of CARE criteria [17]. The recruitment of study subjects was carried out using a nonrandomized and consecutive sampling method of up to 23 patients with an age range from 8 to 16 years old. Parents and/or legal guardians gave their informed consent to the reporting of this case series study.

The inclusion criteria consisted of the paediatric population who received surgical treatment for SO. The exclusion criteria were subjects whose parents and/or legal guardians did not understand the explanations or did not consent to enrolment into the study. 

### 2.2. Procedure

**Figure 1:**
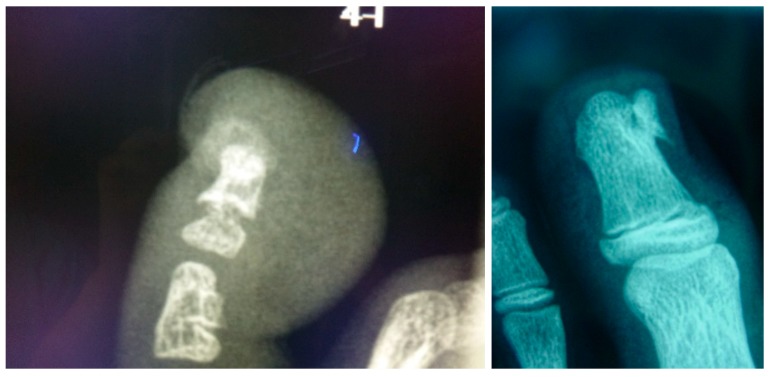
Pre-surgery treatment, clinical appearance and X-ray images for one of the selected cases.

**Figure 2:**
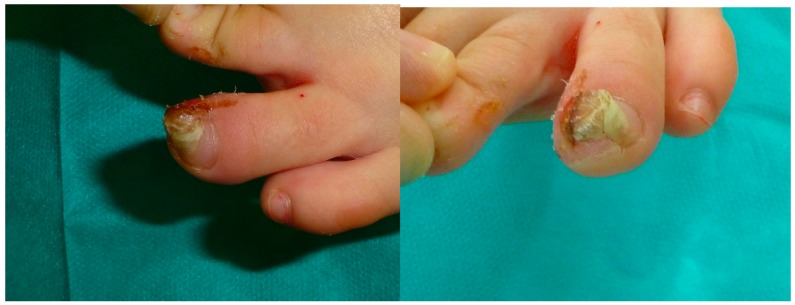
Surgical procedure and clinical outcome of a case with subungual osteochondroma. Preoperative appearance of the lesion. Appearance immediately after the distal wing block anesthesia. Note the subungual mass.

Firstly, radiological features were considered to confirm the presence of SO (Figure 1). The technique followed in the study was performed by the same surgical team according to Valero’s et al. technique [18], which involved the digital nerve-block of the toe with mepivacaine at 1% (Figure 2). A tourniquet was applied at the base of the toe to generate an adequate homeostasis. 

**Figure 3:**
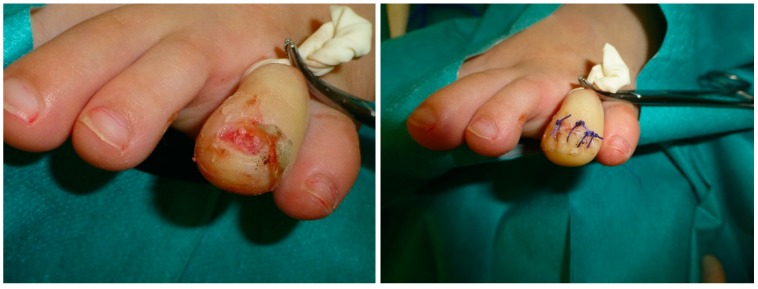
Curettage and removed subungual mass. Destruction of the nail bed after completion of the excision and curettage of the lesion. The nail plate was then debrided of all peripheral tissue and cleansed. The nail plate was sutured back into place, proximally and distally.

Second, the nail is separated from the nailbed with an elevator; we prefer to keep the nail in its nail matrix, if possible. The nailbed is opened using a scalpel’s blade No.15. The nail bed is separated from the bone plane with the scalpel’s blade or elevator to clearly expose the osteochondroma and its limits with the distal phalanx (Figure 3).

**Figure 4:**
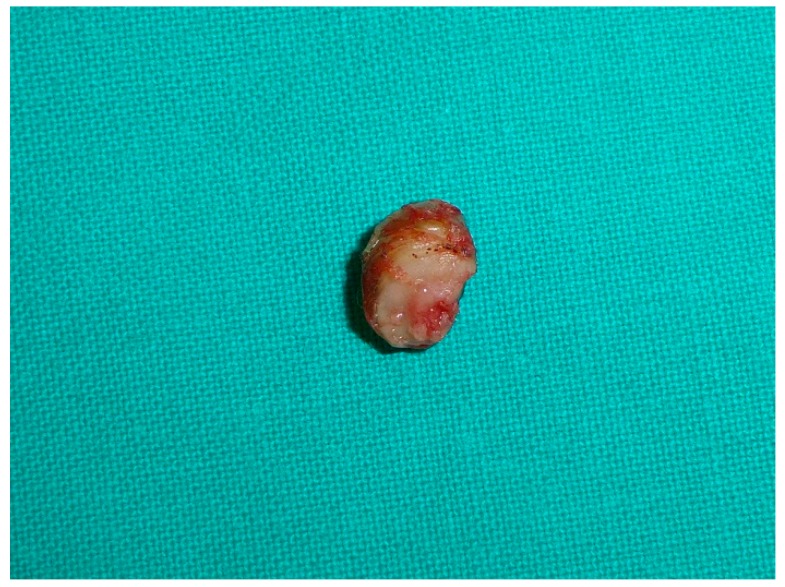
Note subungual osteochondroma mass.

A small osteotome was used to remove the SO from the phalanx. The wound was irrigated with normal saline. The nail bed could then be approximated and repaired with a 4/0 or 5/0 polyglactin absorbable suture. The nail plate was debrided of all peripheral tissue and cleansed (Figure 3). The nail plate was distally sutured back into place. The tourniquet was removed, and a dressing with gauze was placed. The patient returned for examination at 48–72 h. If the sutures did not disappear, they were removed at two weeks after surgery, and finally the SO lesion was considered in histopathological analysis (Figure 4) [11].

### 2.3. Sociodemographic Characteristics 

Socio-demographic data included the age (years), sex (male or female), height (cm), weight (kg) and body mass index (BMI; kg/cm^2^) of the paediatric population diagnosed with SO [18].

### 2.4. Outcome Measurements

Clinical features comprised the side of the affected lower limb (left or right), location (first, second, third, fourth or fifth toe), tumour presence (yes or no), pain presence (yes or no) and nail lift (yes or no) before surgery, as well as recurrence (yes or no) and adverse effects (no adverse effects, pain presence or ulceration) at one month after intervention. New controls are performed at the third and sixth month in which the patients are discharged. All of the patients were also reviewed one year after surgery.

### 2.5. Ethical Considerations

The research was approved by the Research and Ethics Committee of the Universidade da Coruña (Spain), (registry approval date: 07 May 2014). All parents and/or legal guardians gave written, informed consent. In addition, the ethical standards for experimentation in humans were respected as described in the Declaration of Helsinki [19].

### 2.6. Statistical Analysis

Statistical analyses were performed using the version 24.0 of the Statistical Package for Social Science (SPSS Statistics for Windows, IBM Corp, Armonk, NY, USA) considering statistically significant differences for a *p*-value < 0.05 using a 95% confidence interval (CI).

Quantitative data normality was analysed by the Shapiro–Wilk test. Parametric data were described as mean ± standard deviation (SD) and range (minimum–maximum). Non-parametric data were described as median ± interquartile range (IR) and range (minimum–maximum). Statistically significant differences between male and female participants were analysed by the Student *t*-test for independent samples for parametric quantitative data and the Mann–Whitney *U* test for non-parametric quantitative data.

Categorical data were described by frequencies (*n*) and percentages (%). Statistically significant differences between male and female participants were analysed by the Fisher exact test for dichotomous variables and the Chi-squared (χ^2^) test for polytomous variables.

## 3. Results

### 3.1. Sociodemographic Characteristics

A case series of 23 paediatric patients with SO who received surgical treatments completed the study course, including 13 (56.5%) male and 10 (43.5%) female participants with an age mean ± SD of 11.13 ± 2.11 years old and range from 8 to 16 years old. Statistically significant differences (*p* < 0.05) were shown by sex distribution indicating older age and higher height for male participants compared to female participants. The socio-demographic characteristics of the research population are presented in Table 1. 

### 3.2. Clinical Features before Intervention

According to Table 2, the most frequent locations of SO were the first toe (86.8%) and the right lower limb (56.5%). In addition, the presence of the tumour, pain and nail lift showed a prevalence of 91.3%, 69.5% and 47% of the study sample, respectively. There were not statistically significant differences by sex distribution (*p* > 0.05).

### 3.3. Clinical Features at 1 Month after Intervention

Regarding Table 3, the most frequent adverse effect at one month after surgery was pain presence (69.5%). In addition, one case (4.4%) presented ulceration. Only one patient (4.4%) suffered from recurrence, with a new tumour that required a new surgery consisting of amputation of the distal phalangeal, which resolved this condition. There were not statistically significant differences by sex distribution (*p* > 0.05).

## 4. Discussion

The aim of our research was to describe a case series treated by surgical treatment of SO, showing that only one recurrence episode was shown, as well as most cases being resolved. The most prevalent minor adverse effect was pain presence. Finally, sex distribution did not influence the case series’ findings.

The results of the present study showed that excisional surgery has been used as a treatment for SO in paediatric populations as an advantageous intervention, because it may be considered as a rapid treatment with minor adverse effects [11,18,20].

Currently, few studies have described this pathology in paediatric populations [6,7], and in other localizations, according to the prior literature. In addition, less than 15% of SO were located out of the hallux region, in line with a prior study [21]. 

According to these results, another finding was that lesions located on second toes to fifth toes protruded the top of the toe and also caused some damage to the nail plate.

Research with six subjects with SO showed that the mean age in patients who suffered this injury was 10 years, the women/men ratio was 1/1, and the presence of the injury before the diagnosis was two years [22]. Although the present research was carried out in paediatric populations, the results of most studies were in adult populations, and this may modify the presence of the injuries due to the growing period of the bone.

Regarding sex condition, in other research, women accounted for all SO cases [22]. In most cases, the SO affected the hallux. In line with our results, Göktay et al. [23] showed that the majority of SO subjects were women. 

The most frequently reported complication was dystrophic nails and, regarding the ethology of this disorder, trauma was reported as the first cause [23].

The contribution of our research provided a very high satisfaction rate of results, in concordance with other previous studies with similar findings and analogue therapies [1,3,6,10,20].

This intervention may be considered as a simple surgical procedure applicable in the ambulatory setting of the daily practice for a specialized assistance service, and may be generalizable to adult patients, as proposed by other authors [3,23].

Regarding the results of our study, SO may be treated with excellent results using excisional surgery as a possible easy and effective treatment removing the lesion, preventing recurrence and with minor adverse effects. These advantages should be considered for future studies in order to determine the effectiveness of this therapy. In addition, this intervention only removed the lesion; scratching the base may lead to a high recurrence degree.

Regarding the diagnosis of SO, the clinical differential diagnosis included nail disorders, like onicodistrophia, glomus tumours, or subungual malignant melanomas [3,18].

There are several limitations in this research that should be acknowledged. In the first place, future studies should compare excisional intervention and other therapies using a double-blinded, controlled study design. In second place, a larger and more diverse (subjects from different countries) sample size would be beneficial to improve the strength of the study and finally identify more subcategories related with this condition. This highlights the need for further research on SO, recognized as a disorder, and the need for continued research into this common surgical challenge.

## 5. Conclusions

This novel study showed that surgery treatment for SO in paediatric populations presented a very low recurrence degree with minor adverse effects and without differences by sex distribution. Thus, further randomized clinical trials should be carried out in order to determine the effectiveness of this intervention in this special population.

## Figures and Tables

**Table 1:** Socio-demographic characteristics of the sample population.

	Total Group (*n* = 23)	Male (*n* = 13)	Female (*n* = 10)	*p*-Value Male vs. Female
Age (years)	11.13 ± 2.11	11.92 ± 2.10	10.10 ± 1.72	0.037 *
	(8.00–16.00)	(9.00–16.00)	(8.00–12.00)	
Weight (kg)	38.60 ± 14.10	42.80 ± 25.15	37.15 ± 13.84	0.186 ^†^
	(36.72–47.69)	(31.90–77.55)	(27.90–48.00)	
Height (m)	1.45 ± 0.12	1.50 ± 0.12	1.39 ± 0.09	0.020 *
	(1.25–1.74)	(1.34–1.74)	(1.25–1.52)	
BMI (kg/m^2^)	18.77 ± 3.16	18.57 ± 4.74	18.79 ± 1.95	0.927 ^†^
	(16.38–25.53)	(16.38–25.53)	(17.03–21.24)	

*Abbreviations*: BMI, body mass index; IR, Interquartile range; SD, standard deviation. * Mean ± SD, range (minimum–maximum), and Student *t*-test for independent samples were applied. ^†^ Median ± IR, range (minimum–maximum), and Mann–Whitney *U* test were applied. For all analyses, *p*-value < 0.05 (with a 95% confidence interval) was considered as statistically significant.

**Table 2:** Clinical features of the sample population before intervention.

		Total Group *n* (%) (*n* = 23)	Male *n* (%) (*n* = 13)	Female *n* (%) (*n* = 10)	*p*-Value Male vs. Female
Lower limb side	Left	10 (43.5%)	5 (38.5%)	5 (50%)	0.685 *
	Right	13 (56.5%)	8 (61.5%)	5 (50%)	
Toe location	1st	20 (86.8%)	12 (92.3%)	8 (80%)	0.325 ^†^
	2nd	1 (4.4%)	1 (7.7%)	0 (0%)	
	3rd	0 (0%)	0 (0%)	0 (0%)	
	4th	1 (4.4%)	0 (0%)	1 (10%)	
	5th	1 (4.4%)	0 (0%)	1 (10%)	
Tumour presence	Yes	21 (91.3%)	12 (92.3%)	9 (90%)	1.000 *
	No	2 (8.7%)	1 (7.7%)	1 (10%)	
Pain presence	Yes	16 (69.5%)	9 (69.2%)	7 (70%)	1.000 *
	No	7 (30.5%)	4 (30.8%)	3 (30%)	
Nail lift (yes/no)	Yes	11 (47.8%)	6 (46.1%)	5 (50%)	1.000 *
	No	12 (52.2%)	7 (53.9%)	5 (50%)	

* Frequency, percentage (%), and Fisher exact test were applied. ^†^ Frequency, percentage (%), and Chi-Square (χ^2^) test were applied. For all analyses, *p*-value < 0.05 (with a 95% confidence interval) was considered as statistically significant.

**Table 3:** Clinical features of the sample population at one month after intervention.

		Total Group *n* (%) (*n* = 23)	Male *n* (%) (*n* = 13)	Female *n* (%) (*n* = 10)	*p*-Value Male vs. Female
Adverse effects	No	6 (26.1%)	4 (30.8%)	2 (20%)	0.523 ^†^
	Pain	16 (69.5%)	8 (61.5%)	8 (80%)	
	Ulceration	1 (4.4%)	1 (7.7%)	0 (0%)	
Recurrence	Yes	1 (4.4%)	1 (7.7%)	0 (0%)	1.000 *
	No	22 (95.6%)	12 (92.3%)	10 (100%)	

* Frequency, percentage (%), and Fisher exact test were applied. ^†^ Frequency, percentage (%), and Chi-Square (χ^2^) test were applied. For all analyses, *p*-value < 0.05 (with a 95% confidence interval) was considered as statistically significant.
